# Crystal Structure of LSD1 in Complex with 4-[5-(Piperidin-4-ylmethoxy)-2-(*p*-tolyl)pyridin-3-yl]benzonitrile

**DOI:** 10.3390/molecules23071538

**Published:** 2018-06-26

**Authors:** Hideaki Niwa, Shin Sato, Tomoko Hashimoto, Kenji Matsuno, Takashi Umehara

**Affiliations:** 1 Laboratory for Epigenetics Drug Discovery, RIKEN Center for Biosystems Dynamics Research (BDR), 1-7-22 Suehiro-cho, Tsurumi, Yokohama 230-0045, Japan; hideaki.niwa@riken.jp (H.N.); shin.sato@riken.jp (S.S.); 2 Department of Chemistry and Life Science, School of Advanced Engineering, Kogakuin University, 2665-1 Nakano, Hachioji, Tokyo 192-0015, Japan; hashimoto@kanagawa-iri.jp (T.H.); matz@cc.kogakuin.ac.jp (K.M.)

**Keywords:** chromatin, epigenetics, histone demethylase, protein-inhibitor complex, crystal structure

## Abstract

Because lysine-specific demethylase 1 (LSD1) regulates the maintenance of cancer stem cell properties, small-molecule inhibitors of LSD1 are expected to be useful for the treatment of several cancers. Reversible inhibitors of LSD1 with submicromolar inhibitory potency have recently been reported, but their exact binding modes are poorly understood. In this study, we synthesized a recently reported reversible inhibitor, 4-[5-(piperidin-4-ylmethoxy)-2-(*p*-tolyl)pyridin-3-yl]benzonitrile, which bears a 4-piperidinylmethoxy group, a 4-methylphenyl group, and a 4-cyanophenyl group on a pyridine ring, and determined the crystal structure of LSD1 in complex with this inhibitor at 2.96 Å. We observed strong electron density for the compound, showing that its cyano group forms a hydrogen bond with Lys661, which is a critical residue in the lysine demethylation reaction located deep in the catalytic center of LSD1. The piperidine ring interacts with the side chains of Asp555 and Asn540 in two conformations, and the 4-methylphenyl group is bound in a hydrophobic pocket in the catalytic center. Our elucidation of the binding mode of this compound can be expected to facilitate the rational design of more-potent reversible LSD1 inhibitors.

## 1. Introduction

Methylation of histone lysine residues modulates gene expression, activating or inactivating it depending on the lysine site and degree of methylation [1]. Lysine-specific demethylase 1 (LSD1, also known as KDM1A) was the first histone demethylase to be reported, and its discovery proved that histone lysine methylation is a dynamic process regulated by methyltransferases and demethylases [2]. The biochemical properties and biological roles of LSD1 have been extensively investigated. LSD1 demethylates mono- and dimethylated Lys4 of histone H3 (H3Lys4), using flavin adenine dinucleotide (FAD) as a coenzyme [3]. The enzyme also demethylates H3Lys9 when bound to other proteins (e.g., the androgen receptor) or when expressed in its shortest isoform [4,5,6]. The catalytic domain is similar in its amino acid sequence and 3D structure to lysine-specific demethylase 2 (LSD2, also known as KDM1B) and other families of FAD-dependent enzymes, such as monoamine oxidases (MAOs) and polyamine oxidases (PAOs). However, LSD1 is unique in that its catalytic domain contains an elongated Tower domain where the REST corepressor 1 protein (CoREST) binds and presumably mediates LSD1 binding to a nucleosome (reviewed in [7]). LSD1 is highly expressed in several cancers and is involved in maintaining the stem cell properties of cancer cells, such as those involved in acute myeloid leukemia (AML) and glioblastoma [8,9,10]. Therefore, LSD1-selective inhibitors are expected to serve as therapeutic agents for these diseases.

In the early stages of the development of LSD1 inhibitors, *trans*-2-phenylcyclopropylamine (2-PCPA, or tranylcypromine), a MAO inhibitor, was used as a molecular scaffold. Compounds based on this scaffold are irreversible inhibitors that covalently bind to FAD. Information obtained from crystal structures of LSD1 or the LSD1•CoREST complex, bound to 2-PCPA and its derivatives (review [7]) has been used to develop potent and selective inhibitors of LSD1 [11,12], some of which are currently being tested in early-phase clinical trials [7,13]. In contrast, progress toward the development of reversible small-molecule inhibitors that noncovalently bind to LSD1 has been slow, and inhibitory potencies have remained in the micromolar range, possibly because finding a small molecule that binds strongly to the spacious cavity of LSD1, which accommodates the histone H3 N-terminal tail peptide, is difficult. However, several kinds of reversible inhibitors with submicromolar inhibitory potency have recently been reported [7,14]. Development of reversible LSD1 inhibitors may be beneficial in drug development to avoid potential risks that irreversible inhibitors would cause through reactive metabolites, such as an immune response to the adducted protein and an unpredictable idiosyncratic toxicity.

Although many researchers working on the development of reversible LSD1 inhibitors have performed in silico docking experiments, studies involving structural methods such as X-ray crystallography are limited in number. Mattevi and Mai et al. have demonstrated that LSD1 is inhibited by the antibiotics polymyxins B and E, as well as the histone methyltransferase G9a inhibitor E11 [15]. The crystal structures of LSD1•CoREST bound to these compounds indicate that the polymyxin molecules bind to the negatively charged entrance of the substrate-binding cavity of LSD1 in multiple rotated orientations [15]. E11 also binds to the cavity entrance, but in a stack consisting of five to seven molecules of the inhibitor. Recently, Vianello and Sartori et al. [16,17] determined the crystal structure of LSD1•CoREST in complex with a reversible inhibitor that was selected by high-throughput screening of a chemical library. On the basis of the structure, these investigators developed reversible LSD1 inhibitors with single-digit nanomolar IC_50_ values [17]. The binding modes of the newly developed inhibitors were validated on the basis of crystal structures of their complexes with LSD1•CoREST. Finally, the crystal structure of LSD1•CoREST with noncovalently bound tetrahydrofolate has been reported, although tetrahydrofolate had no effect on the activity of LSD1 in an in vitro biochemical assay [18].

**Figure 1:**
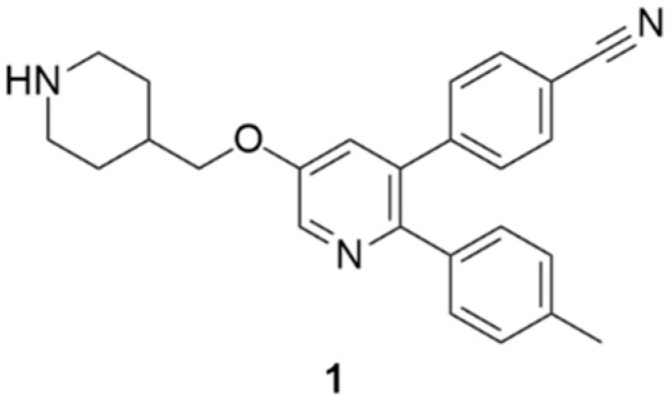
Chemical structure of **1** [19].

In this paper, we present the crystal structure of LSD1•CoREST in complex with 4-[5-(piperidin-4-ylmethoxy)-2-(*p*-tolyl)pyridin-3-yl]benzonitrile (**1**, Figure 1), which was recently reported to be a reversible LSD1 inhibitor with a *K*_i_ of 29 nM [19]. The crystal structure presented here demonstrates that **1** binds in a novel mode that was not fully predicted in the in silico docking study described in the original publication.

## 2. Results

### 2.1. Overall Structure of LSD1•CoREST in Complex with **1**

**Figure 2:**
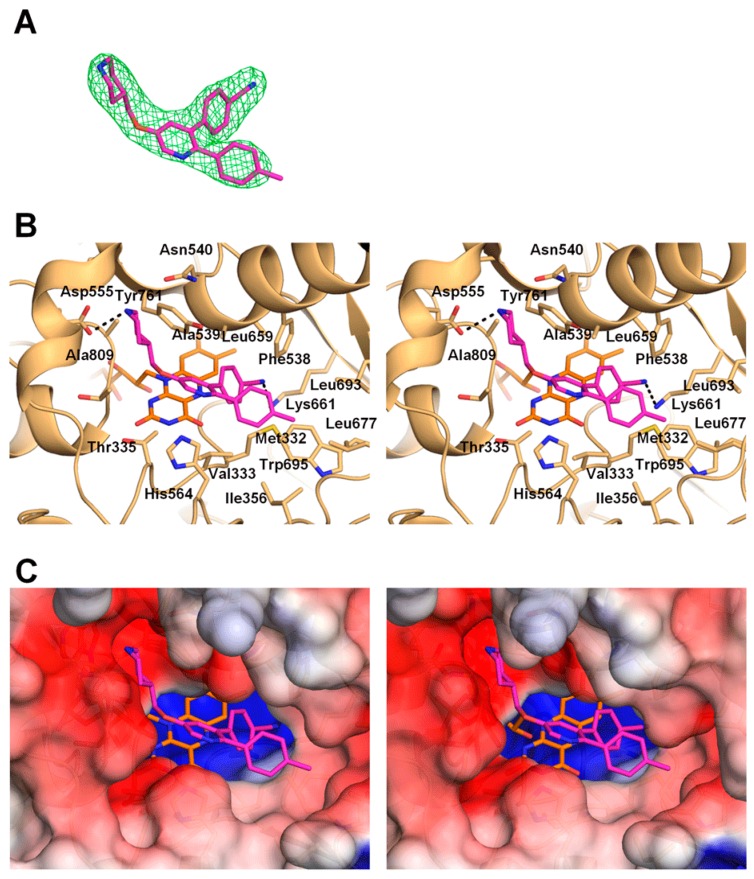
Crystal structure of LSD1 bound to **1**. (**A**) Electron density of **1**, shown as a stick model (magenta) with an omit *mF*_o_-*DF*_c_ electron density map contoured at 2.5 σ (green mesh). (**B**) Stereoview of the binding mode of **1**. LSD1, FAD and **1** are shown in brown, orange, and magenta, respectively. Hydrogen bonds are indicated by dashed black lines. (**C**) Stereoview of an electrostatic surface potential model of the **1**-binding site of LSD1.

Crystals of LSD1•CoREST in complex with **1** were obtained by the soaking method. X-ray diffraction data collected at a synchrotron beamline were processed to a resolution of 2.96 Å (Table 1). The electron density map after molecular replacement clearly revealed the bound inhibitor, which has three branches extending from the pyridine ring at the center of the molecule: a 4-piperidinylmethoxy group, a 4-methylphenyl group, and a 4-cyanophenyl group. Because the 4-piperidinylmethoxy and 4-methylphenyl groups extend linearly from the 5- and 2-positions of the pyridine ring, respectively, and the 4-cyanophenyl group protrudes obliquely from the 3-position, the molecule could be placed into the electron density map with an unequivocal orientation (Figure 2A). Binding of **1** to LSD1 did not affect the overall structure of LSD1•CoREST, except for the positions and orientations of several residues in the catalytic cavity of LSD1, as described below.

### 2.2. Interactions between LSD1 and ***1***

In the crystal structure, the pyridine ring of **1** is located in the middle of the substrate-binding cavity of LSD1, with the center of the ring at 6 Å above the plane of the isoalloxazine ring of FAD. Distances described herein are approximate as observed in the 2.96 Å crystal structure. The pyridine ring is sandwiched between the side chain of Ala539 (4.1 Å away) and the side chains of Thr335 and His564 (4.2 and 4.0 Å away, respectively). The pyridine ring is oriented such that the nitrogen atom is located at the entrance to the binding cavity (on the opposite side of the ring from the FAD molecule) and does not directly interact with any of the atoms of the protein (Figure 2B).

The piperidine ring of **1** is located in a negatively charged pocket formed by the side chains of Asn540 and Asp555 and the main-chain carbonyl groups of surrounding residues (including Ala539 and Ala809; Figure 2B,C). The structure depicted in the figure shows the piperidine ring in a chair conformation with the nitrogen atom forming a 3.3 Å hydrogen bond with one of the side-chain oxygen atoms of Asp555. However, the precise conformation of the piperidine ring could not be determined from the electron density map at the 2.96 Å resolution. If the piperidine nitrogen atom were flipped, it could form a 3.3 Å hydrogen bond with the side-chain oxygen atom of Asn540. Therefore, it is plausible to assume that the piperidine ring can change its conformation to form a hydrogen bond with the side chain of either Asn555 or Asn540. The linker carbon atom and the oxygen atom of the 4-piperidinylmethoxy group are located in the middle of the cavity and do not interact directly with any of the protein atoms.

The 4-methylphenyl group resides in a rather large hydrophobic pocket in the LSD1 substrate-binding cavity. The bottom of the pocket is formed by Val333, Phe538, and Trp695, and the wall is formed by Ile356, Leu677, and Leu693 (Figure 2B,C). The methyl group is tucked into the space between Trp695 and Leu677, 3.3 Å above the Trp695 ring plane and 3.9 Å from the side chain of Leu677.

The 4-cyanophenyl group is bound deep in the substrate-binding cavity, buried under the above-mentioned hydrophobic pocket containing the 4-methylphenyl group (Figure 2B,C). The 4-cyanophenyl group sits in a hydrophobic pocket formed by Met332, Val333, Phe538, Lys661, Leu659, and Tyr761 and is located near the FAD molecule, with the cyano group nitrogen atom 3.9 Å from the closest atom (C6) of the isoalloxazine ring. Importantly, the lone-pair electrons on the nitrogen atom form a 2.9 Å hydrogen bond with the side-chain nitrogen of Lys661.

### 2.3. Comparison of the LSD1•CoREST•1 Structure with that of LSD1•CoREST-Bound H3 Peptide

**Figure 3:**
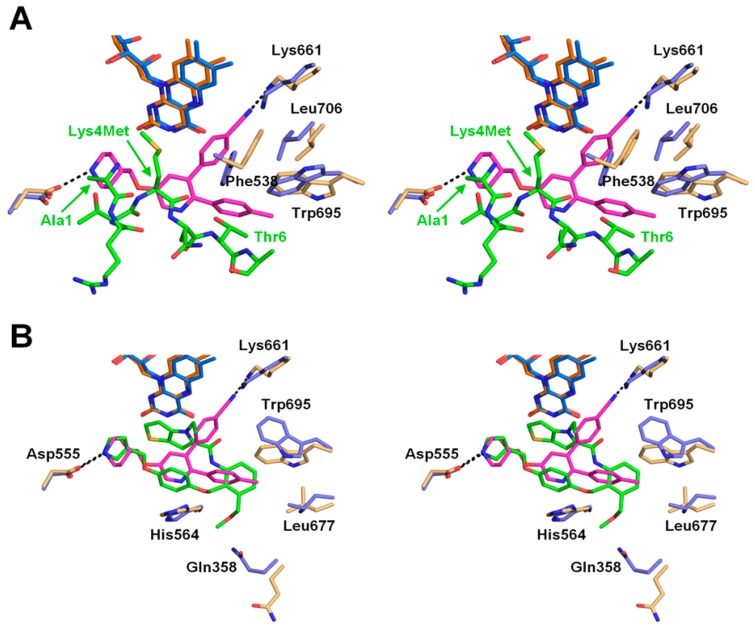
Structural comparison of **1** with a substrate peptide and another reversible inhibitor in stereoview. (**A**) Superimposition with the H3Lys4Met peptide (1–21). (**B**) Superimposition with compound **49** in [17]. For clarity, both view angles are different from that in Figure 2. LSD1, FAD, and **1** are depicted as described in Figure 2B. In (A), LSD1, FAD, and H3Lys4Met in the structure of LSD1•CoREST in complex with H3Lys4Met (PDB ID: 2V1D) [20] are depicted in light blue, dark blue, and green, respectively. The residues of the H3Lys4Met peptide (1–21) that overlap with or are in close proximity to **1** are labeled in green. The LSD1 residues that undergo positional shifts upon binding of **1** are labeled in black. In (**B**), LSD1 and FAD in LSD1•CoREST in complex with **49** (PDB ID: 5LHH) [17] are colored in the same scheme for the H3Lys4Met complex in (**A**), and **49** in green. Selected key residues around the compounds are shown with black labels.

We superimposed the structure of LSD1•CoREST bound to **1** with the structure of the protein complex bound to the H3Lys4Met peptide (1–21) (PDB ID: 2V1D) [20], in which Lys4 of the histone peptide was mutated to methionine (Figure 3A). The comparison demonstrated that the central pyridine ring of **1** occupies the same position occupied by the main-chain atoms of H3Lys4Met. The piperidine ring of **1** is located at the H3Ala1 position, and the 4-methylphenyl ring is located near the position of the side chain of H3Thr6. The superimposition also shows the structural alteration that occurs upon binding of **1**. Specifically, the side chain of Trp695 changes its conformation to accommodate the 4-methylphenyl group of **1**. The χ_1_ angle of the side chain of Phe538 rotates by 41° to accommodate both the 4-methylphenyl and the 4-cyanophenyl groups. In addition, the side chain of Leu706, which is not in close proximity to the inhibitor, changes its conformation to avoid a clash with the dislocated side chain of Phe538. Finally, the side chain of Lys661 also changes its conformation, to avoid a direct clash with the 4-cyano group, and forms a hydrogen bond with the cyano group nitrogen atom.

## 3. Discussion

In this study, we investigated the binding mode of 4-[5-(piperidin-4-ylmethoxy)-2-(*p*-tolyl)pyridin-3-yl]benzonitrile (**1**) to LSD1 by means of X-ray crystallography. The crystal structure, which clearly reveals the electron density of **1**, shows that each of the three branches extending from the central pyridine ring of 1 is anchored to a separate binding pocket in the spacious substrate-binding cavity of LSD1. This binding mode is stabilized by both polar and nonpolar interactions, which explains the high inhibitory potency of **1**.

The piperidine amine interacts with the carboxylate group of Asp555 and the amide oxygen of Asp540, and occupies the same site as the N-terminus of the histone H3 peptide (Figure 3A). This site is also used by other inhibitors that bind noncovalently [15,17]. Specifically, the pyrrolidine rings of recently reported reversible LSD1 inhibitors [17], including compound **49** (PDB ID: 5LHH; Figure 3B), also interact with the carboxylate group of Asp555 by occupying almost the same position as that occupied by the piperidine ring of **1**. The hydrophobic pocket where the 4-methylphenyl group of **1** binds is also used by the recently reported inhibitors [15,17] (Figure 3B). The cyano group in the 4-cyanophenyl moiety of **1** forms a hydrogen bond with the side chain of Lys661, which is a key residue in the catalytic reaction of LSD1, forming a water-mediated hydrogen bond with the N5 atom of FAD in the structure of the ligand-free LSD1 [21]. The 4-cyanophenyl ring replaces the water molecule to form a hydrogen bond with Lys661. No other LSD1 inhibitors, either covalent or noncovalent, that interact directly with Lys661 were hitherto known in the Protein Data Bank (PDB) (see the discussion on GSK-690 below).

The importance of structural studies cannot be overemphasized, as indicated by the fact that the binding mode of inhibitor **1** revealed by our study differs from that suggested by an in silico docking study [19]. Although the docking study indicated that the piperidine interacts with Asp555, as was observed in our crystal structure, the pyridine ring in the in silico study was rotated by about 180° relative to its position in the crystal structure, with its nitrogen on the FAD side of the pyridine ring. As a result, the positions of the 4-methylphenyl and 4-cyanophenyl rings suggested by the in silico study were swapped compared with those in our crystal structure. In the docking model, the hydrophilic and hydrophobic interactions between the pyridine ring and Tyr761 and FAD may have contributed to the suggested orientation. In any case, hydrogen bonding between the cyano group and Lys661 should stabilize the binding of **1** in the orientation as observed in the crystal structure. Because the actual binding mode of **1** to LSD1 has now been elucidated, the structure-activity relationships described in the original report [19] on the activity of **1** can be interpreted based on the crystal structure. The key role of the cyano group in improving the potency of the inhibitor can now be understood to be due to its interaction with Lys661, which is critically important for lysine demethylation. Replacement of the methyl group in the 4-methylphenyl moiety with a CF_3_ group, an OCF_3_ group, or an isopropyl group did not substantially reduce the potency of the inhibitor, a result that is consistent with the fact that the methyl group lies in a rather large hydrophobic pocket.

The present crystal structure will be informative for future structure-based inhibitor design. For example, it is interesting to diversify each branch of **1** based on the structure. A trial to increase interactions with LSD1 by referring to the binding modes of other reversible inhibitors (Figure 3B) would be another option. In addition, protrusion to a nearby concave area, such as the one to which Thr3 of the H3Lys4Met peptide binds (bottom left pocket in Figure 2C), may also be effective for better potency. It should be noted that **1** is structurally similar to another reversible LSD1 inhibitor, GSK-690 (also known as GSK-354). The only difference is that GSK-690 bears a pyrrolidine ring in place of the piperidine ring of **1** [14]. Because of this similarity, the binding mode of GSK-690 is expected to be the same as that of compound **1** presented in this study. A crystal structure of GSK-690 bound to LSD1 is referred to in one report [22], but to our knowledge, structural data for the LSD1•GSK-690 complex are not available in the public domain.

In summary, we determined the crystal structure of LSD1•CoREST in complex with **1**, a previously reported LSD1 inhibitor [19]. The crystal structure demonstrates that the piperidine ring binds to the negatively charged pocket, interacting alternately with Asp555 and Asn540. The 4-methylphenyl group binds to a hydrophobic pocket, and the 4-cyanophenyl group binds deep in the substrate-binding cavity of LSD1 and interacts directly with Lys661. The binding mode revealed by this X-ray crystallography study can be expected to serve as a basis for the development of more-potent inhibitors for LSD1.

## 4. Materials and Methods

### 4.1. General Information

^1^H-NMR spectra were recorded with a JEOL (Akishima, Japan) JNM-ECZ400S NMR spectrometer. Chemical shifts (δ) are expressed in parts per million (ppm) relative to the internal standard tetramethylsilane (s, singlet; d, doublet; m, multiplet; br, broad; brm, broad multiplet). Electrospray ionization mass spectra were recorded on a JEOL JMS-GCmate II spectrometer.

### 4.2. Synthesis of ***1***

Compound **1** (Figure 1) was synthesized as described previously [19]. ^1^H-NMR (400 MHz, DMSO-*d*_6_): δ 9.16 (1H, br), 8.89 (1H, br), 8.48 (1H, d, *J* = 2.8 Hz), 7.82 (2H, d, *J* = 8.2 Hz), 7.62 (1H, s), 7.42 (2H, d, *J* = 8.2 Hz), 7.13–7.08 (4H, m), 4.09 (2H, d, *J* = 6.3 Hz), 3.29 (2H, brm), 2.91 (2H, brm), 2.28 (3H, s), 2.13 (1H, br), 1.94 (2H, brm), 1.54 (2H, brm). EI-MS *m/z*: 383.

### 4.3. Protein Preparation and Crystallization

Human LSD1 (residues 172–833), human CoREST (residues 308–440), and the LSD1•CoREST complex were prepared as described previously [23]. Crystals of LSD1•CoREST were grown also as described previously [23]. Briefly, crystallization was performed by the hanging drop method at 20 °C, by mixing an LSD1•CoREST solution with a reservoir solution containing 100 mM *N*-(carbamoylmethyl)iminodiacetic acid (pH 5.5) and 1.28 M potassium sodium tartrate. Crystals were soaked overnight in the reservoir solution containing 2 mM **1** and 10% glycerol and were then flash-cooled in liquid nitrogen for data collection. The reservoir solution containing 20% glycerol was used as the cryoprotectant.

### 4.4. X-ray Diffraction Data Collection and Structure Determination

The diffraction data were collected at BL26B2 of SPring-8 (Sayo, Hyogo, Japan). The data were processed with XDS [24] and programs in the CCP4 suite [25]. Molecular replacement was performed with Phaser [26] using the protein portion of the structure of LSD1•CoREST in complex with an inhibitory peptide (PDB ID: 5H6Q) as the search model [23]. Initial coordinates and parameters for **1** were generated with Grade [27] from the SMILES (Simplified Molecular Input Line Entry System) string of the inhibitor. The structure was refined using PHENIX [28] with manual model building using Coot [29]. The model quality was assessed using MolProbity [30]. Data collection and refinement statistics are summarized in Table 1. The molecular models were drawn with PyMOL [31], and the electrostatic surface potential was calculated with the APBS plugin [32] for PyMOL. The coordinates and structure factors have been deposited in the PDB under accession number 5YJB.

## Figures and Tables

**Table 1:** Crystallographic data collection and refinement statistics.

PDB ID	5YJB
**Data Collection**	
Beamline	SPring-8 BL26B2
Wavelength (Å)	1.000
Space group	*I*222
**Unit Cell Dimensions**	
*a*, *b*, *c* (Å)	121.1, 179.6, 235.0
*α*, *β*, *γ* (°)	90, 90, 90
Resolution (Å)	48.82−2.96 (3.05−2.96) *
No. of unique reflections	53,652
Completeness (%)	100 (100)
Multiplicity	7.5 (7.6)
Mean *I*/*σ* (*I*)	16.2 (1.7)
*R*_sym_ (%)	11.4 (142)
*R*_pim_ (%)	4.4 (54.8)
CC_1/2_	0.999 (0.747)
**Refinement**	
*R*_work_ (%)	18.9
*R*_free_ (%)	21.9
RMSD bond lengths (Å)	0.008
RMSD bond angles (°)	0.945
No. of reflections (total)	53,614
No. of reflections (test set)	1053
**No. of Atoms**	
Total	6313
Protein	6219
FAD	53
Inhibitor	29
Glycerol	12
**Mean *B*-factor (Å^2^)**	
Overall	92.9
Protein	93.2
FAD	67.8
Inhibitor	66.6
Glycerol	82.9
**Ramachandran Plot (%)**	
Favored	96.5
Allowed	3.5
Molprobity clashscore	4.6 (100th percentile)
Molprobity score	1.45 (100th percentile)

* Values in parentheses are for the highest-resolution bin.
